# Dual room‐temperature phosphorescence derived from the reversible homolysis in the high proportion n‐electron organic crystal

**DOI:** 10.1002/smo2.70083

**Published:** 2026-07-21

**Authors:** Junxiang Huang, Dongqian Wang, Dongping Wang, Qichao Yao, Xingyu Huangfu, Guian Yang, Saran Long, Jianjun Du, Jiangli Fan, Xiaojun Peng

**Affiliations:** ^1^ State Key Laboratory of Fine Chemicals Frontiers Science Center for Smart Materials Dalian University of Technology Dalian China; ^2^ Chemistry Analysis & Research Center School of Chemical Engineering Dalian University of Technology Dalian China; ^3^ Shandong Laboratory of Advanced Materials and Green Manufacturing at Yantai Yantai China

**Keywords:** dual room‐temperature phosphorescence, n‐electron, radical ion pair, reversible homolysis

## Abstract

The reversible transformation between organic molecules and radicals is an effective method for realizing dual emissions in a single material. Herein, in this work, we report a high proportion n‐electron [2,2′‐biisoindoline]‐1,1′,3,3′‐tetraone (4A2B) crystal with the reversible homolysis. The crystal exhibits dual room‐temperature phosphorescence (RTP) emissions at 400 nm (*τ* = 114.28 μs) and 575 nm (*τ* = 35.78 μs), originating from the 4A2B molecule and the corresponding radical ion pair generated by reversible homolysis of the weak N–N bond, respectively. Thanks to that, the radical ion pair performed a great photostability (*t*_1/2_ = 1.64 × 10^5^ s) and an outstanding repeatability under 100 times on‐off excitation cycles. The RTP emission of the 4A2B molecule has a strong response on high energy excitation due to the ultrafast intersystem crossing process (1.22 × 10^13^ s^−1^) within the high‐lying excited states. Furthermore, the 4A2B crystal with these two long‐lived RTP emissions is able to be applied in optical anti‐counterfeiting, optical encryption and high‐resolution bioimaging. This research elucidates that the incorporation of organic molecules and radical ion pairs may provide a new method to achieve dual emissions containing RTP emissions, fluorescence or thermally activated delayed fluorescence.

## Graphical abstract

[2,2′‐biisoindoline]‐1,1′,3,3′‐tetraone (4A2B) crystal with high n‐electrons undergoes photo‐induced reversible homolysis resulting in two room‐temperature phosphorescence (RTP). One of the 4A2B molecules responds strongly to 250 nm via fast intersystem crossing (ISC) processes among high‐lying excited states. Another radical ion pair has good photostability and repeatability. This work offers a new guide for multiple emissions, especially RTP.

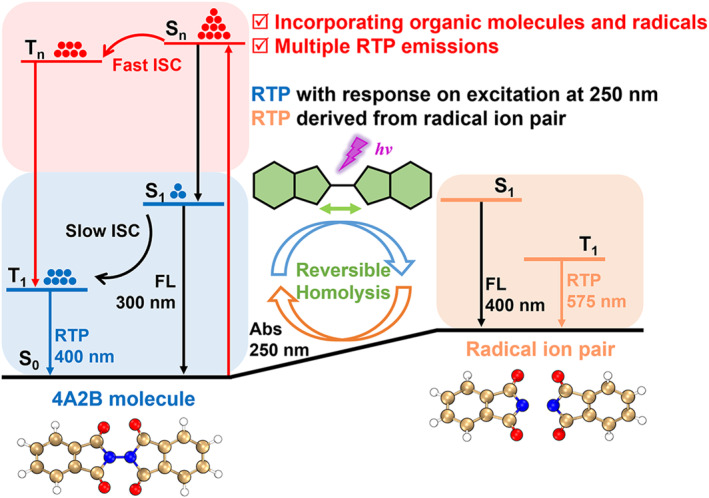

## 1 INTRODUCTION

Room‐temperature phosphorescence (RTP) materials have been widely applied in optical anti‐counterfeiting, optical encryption and high‐resolution bioimaging because of their long‐lived lifetime distinguished from fluorescence.[^1^, ^2^, ^3^, ^4^] Single RTP emission has been studied for almost a century.[^5^, ^6^, ^7^] There are a lot of methods to manipulate RTP emission, such as introducing heavy atom to promote the intersystem crossing (ISC), increasing (intra‐) intermolecular hydrogen bonds or doping in the rigid matrix to stabilize the triplet exciton, and so on.[^8^, ^9^, ^10^, ^11^, ^12^, ^13^, ^14^] Nevertheless, the increasing demand for advanced functional materials has driven research toward multiplexed design. Realizing dual RTP emissions within a single system is a significant challenge and has emerged as a frontier topic in the field. Dual RTP emissions with multi‐scale lifetimes have more complicated mechanisms and more extensive applications.[^15^, ^16^, ^17^, ^18^, ^19^, ^20^, ^21^, ^22^, ^23^, ^24^, ^25^, ^26^, ^27^, ^28^] To date, several mechanisms have been proposed to account for dual RTP emissions, including molecular aggregation, anti‐Kasha's rule and molecular conformation regulation.[^15^, ^16^, ^22^, ^24^, ^26^, ^27^, ^28^] However, the rich intermolecular interactions and long‐lived triplet states inherent to RTP materials may trigger complex transformation of the molecular structure. Understanding the dynamic process behind such transformations is therefore crucial for designing new dual RTP emission materials.^[15]^

Homolysis is a suitable dynamics to realize dual RTP emissions, as it directly cleaves covalent bonds in organic molecules to yield radical species. These two species (radicals and organic molecules) represent two different electronic structures and excited state characteristics corresponding to two different emission pathways. In general, radicals are instable due to their unpaired electrons, even in the case of the persistent organic radicals. For certain optical applications, the long‐term persistence of radicals is not required in the actual operation. For applications such as bioimaging, advanced anti‐counterfeiting and dynamic information encryption, it is often sufficient that radicals are generated selectively during the photoexcitation period, as their brief existence can be precisely synchronized with the emission. This effectively decouples the need for chemical stability from the emissive function. The radical ion pair, constituted of a radical cation and a radical anion, is usually generated by photo‐induced charge transfer processes but is able to recombine when the external energy is removed, which means the reversible formation of the radical ion pair.[^29^, ^30^, ^31^] Thus, the strategic implementation of reversible homolysis provides a versatile and dynamic platform for constructing sophisticated dual RTP emission materials, where the emission can be intricately linked to photo‐induced homolysis and radical dynamics. In contrast to the reported dual RTP emissions strategies, the homolysis pathway generates radical species, rendering the RTP emission amenable to external modulation by magnetic fields.

In this work, we present an organic crystal composed solely of [2,2′‐biisoindoline]‐1,1′,3,3′‐tetraone (4A2B), in which two phthalimide units are linked by a weak N–N single bond. The crystal exhibits dual RTP emissions at 400 nm (*τ* = 114.28 μs) and 575 nm (*τ* = 35.78 μs), originating from the 4A2B molecule and the corresponding radical ion pair generated by reversible homolysis of the weak N–N bond, respectively. The high proportion n‐electron (n/π electron = 1, Supporting Information S1; Figure S1) of the 4A2B molecule enhances the spin–orbit coupling (SOC) effect, thereby promoting both ISC and RTP efficiency. Notably, the rate of ISC from higher‐lying singlet excited states (S_*n*_) reaches 1.22 × 10^13^ s^−1^, significantly exceeding that from S_1_ and rivaling the rate of internal conversion. This rapid ISC pathway leads to a strong RTP response on 250 nm excitation. The formation of the radical ion pair is confirmed by electron paramagnetic resonance (EPR) spectroscopy, with its rise time of 5.29 μs observed in the RTP kinetics further supporting its generation. The radical ion pair demonstrated remarkable photostability (*t*_1/2_ = 1.64 × 10^5^ s) and excellent reproducibility over 100 excitation on‐off cycles, consistent with reversible homolysis. This integration of molecular and radical‐based emissions offers a new design strategy for realizing multi‐emission systems featuring RTP, fluorescence, or thermally activated delayed fluorescence (TADF).

## 2 RESULTS AND DISCUSSION

**FIGURE 1:**
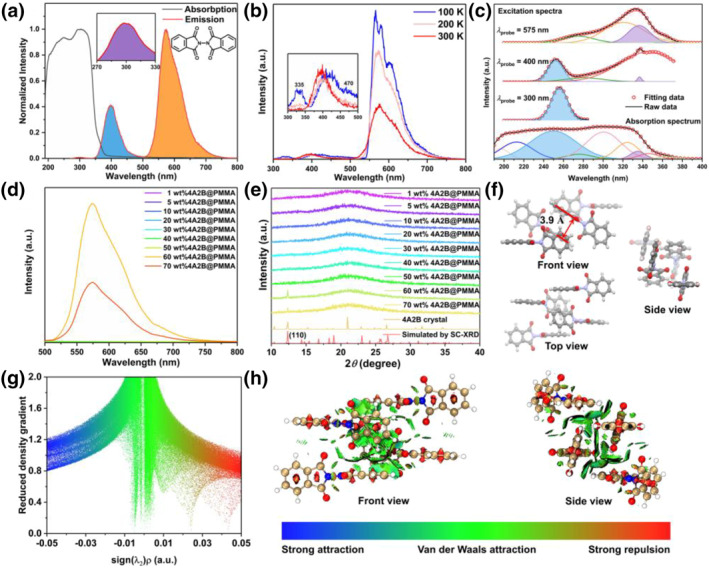
(a) Absorption and PL (*λ*_exc_ = 250 nm) spectra of 4A2B crystal. (b) PL spectra (*λ*_exc_ = 250 nm) of 4A2B crystal at different temperatures. (c) Photoluminescence excitation (PLE) spectra (*λ*_probe_ = 300 nm/400 nm/575 nm) and absorption spectrum of 4A2B crystal after peak fitting, each sub‐peak in the PLE spectra corresponding to a sub‐peak in the absorption spectrum. (d) PL spectra (*λ*_exc_ = 250 nm) of 4A2B in PMMA films with different weight fractions from 1% to 70%. (e) Powder X‐ray diffraction (PXRD) patterns of 4A2B in PMMA films with different weight fractions from 1% to 70% and 4A2B crystal. (f) Crystal structure of 4A2B crystal. (g) Scatter plot and (h) Reduced gradient isosurfaces (*s* = 0.75 a.u.) are colored according to the corresponding values of sign(*λ*_2_)*ρ* by Non‐covalent interactions (NCI) analysis.

The absorption and photoluminescence (PL) spectra of the 4A2B crystal were systematically investigated to elucidate its photophysical properties. As depicted in Figure 1a, the absorption spectrum exhibits two distinct bands at 250 and 300 nm. Specifically, under the excitation at 250 nm, there are two widely‐separated emissions centered at 400 and 575 nm, accompanied by a weak emission near 300 nm. Additional weak emissions centered at 335 and 470 nm emerge at 100 K (Figure 1b), which are attributed to transitions from an unstable excited state (e.g., transition state) or symmetry‐forbidden processes.[^32^, ^33^] The purity of 4A2B crystals, verified by nuclear magnetic resonance spectroscopy and high‐performance liquid chromatography (Supporting Information S1; Figure S2), confirms that none of the observed emissions arise from impurities. Quantum chemistry calculations were performed using the TDDFT to gain a deep understanding of each emission. The “our Own N‐layer Integrated molecular Orbital molecular Mechanics” (ONIOM) model (Supporting Information S1; Figure S3) was used to simulate the environment of the crystal. As shown in Supporting Information S1; Table S1, the TDDFT calculation indicates that the emission at 300 and 400 nm can be assigned to the transition of S_1_→S_0_ (ground state) and T_1_→S_0_ of the 4A2B molecule, but the emission at 575 nm still needs a further study.

To decipher the complex photoluminescence, we analyzed the photoluminescence excitation (PLE) spectra, which allow the grouping of emissions into potential fluorescence‐phosphorescence pairs based on shared excitation features. The PLE spectra were resolved into characteristic peaks corresponding to those in the absorption spectrum, as depicted in Figure 1c. The emissions at 300 nm share a common excitation peak (250 nm, blue peak) with the fluorescence of the 4A2B molecule (400 nm), which identified them as a fluorescence‐phosphorescence pair originating from the 4A2B molecule, consistent with the quantum chemical calculations. In addition, the PLE spectrum probed at 400 nm also shares a common excitation peak (337 nm, violet peak) with the emission at 575 nm. This suggests that the emission at 400 nm may contain a fluorescence component, which could be paired with the 575 nm emission as another fluorescence‐phosphorescence pair. However, the stacking arrangements of molecules may result in aggregates within the crystal. The assignment of second fluorescence‐phosphorescence pair requires a further verification.

To investigate whether there are any aggregate species (e.g., multimers or excimers), we prepared poly(methyl methacrylate) (PMMA) films doped with 4A2B at varying concentrations (1–70 wt%) and examined their photoluminescence and powder X‐ray diffraction patterns (Figure 1d,E). The emission at 575 nm and the diffraction peak corresponding to the (110) crystal plane emerged only in films with high doped concentrations (≥60 wt%). This strongly connects the emission at 575 nm to the crystalline phase. The intermolecular distance within the 4A2B crystal is approximately 4 Å (Figure 1f, lattice parameters are shown in Supporting Information S1; Table S2). Non‐covalent interactions analysis[^34^, ^35^] revealed the low‐density, low‐gradient, green spikes (Figure 1g), which represent Van der Waals attraction corresponding to the green isosurfaces (Figure 1h) among 4A2B molecules. However, TDDFT calculations (Supporting Information S1; Table S1) reveal that the vertical excitation energy of any multimers (dimers, trimers and tetramers, shown in Supporting Information S1; Figure S4) have no difference. This implies that the molecular orbitals of the 4A2B molecule are not altered significantly (Supporting Information S1; Figure S5) and the crystal environment primarily provides the steric hindrance. Besides, excimer usually exhibits three spectroscopic signatures, the red shift of emission, the significant spectral broadening and the absence of vibrational fine spectral structure.[^36^, ^37^] However, there are three sub‐peaks of the emission at 575 nm at 100 K (Figure 1b), revealing the resolved vibrational fine structure, which excludes the presence of excimer in 4A2B crystal.

**FIGURE 2:**
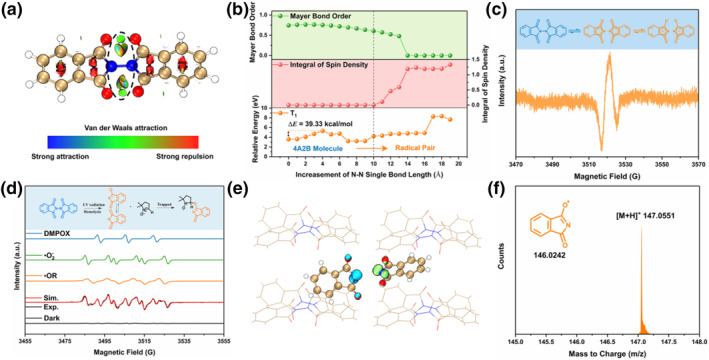
(a) Non‐covalent interactions (NCI) analysis of the 4A2B molecule. (b) Mayer bond order (top), integral of spin‐up (‐down) density (medium) and potential energy surface (PES) of T_1_ (bottom) with N‐N single bond length increasement. The length of N‐N single bond is selected as the only variable parameter for rigid scanning of PES which is extended from 1.37 Å to 2.17 Å with step size of 0.1 Å. (c) electron paramagnetic resonance (EPR) spectra of 4A2B crystal, *g* = 2.0001. (d) EPR and simulated spectra of 4A2B solution, solvent: tert‐butyl benzene, simulation parameters: DMPOX, A_N_ = 14.0844 G, *g* = 2.00515; superoxide radical, A_N_ = 14.1259 G, A_H*β*_ = 11.7171 G, *g* = 2.00531; alkoxy radical, A_N_ = 13.0438 G, A_H*β*_ = 9.97087 G, A_H*γ*_ = 1.39149 G, *g* = 2.0054. (e) The spin density of homolyzed 4A2B molecule at the ground state, blue and green isosurfaces represent the spin‐up and spin‐down density respectively, isovalue = 0.02. (f) High‐resolution mass spectrum of 4A2B solution in positive ion mode with atmospheric pressure chemical ionization (APCI) source.

The bond energy of an N‐N single bond (∼160 kJ/mol) is only about half that of a carbon‐carbon single bond (∼347 kJ/mol).[^38^, ^39^, ^40^, ^41^] In addition, there is repulsion between the two phthalimides demonstrated in Figure 2a. These factors could induce the homolysis of the N‐N single bond with external energy. As illustrated in Figure 2b, the Mayer bond order of the N‐N single bond is 0.74 closed to 1, which denotes a weak single bond feature. Then, the Mayer bond order progressively decreases to zero with increasing bond length, indicating the homolysis of the N‐N single bond. In the meanwhile, the integral of spin‐up (‐down) density increases from 0 to ∼1 corresponding to the generation of radical pair. There is a stable platform observed in the potential energy surface of T_1_ representing the radical pair. The energy gap of homolysis is about 39.33 kcal/mol, which can be satisfied under the photoexcitation. The EPR spectrum (Figure 2c) of the 4A2B crystal denotes a signal of a radical ion pair confirming the homolysis of the N‐N single bond.[^30^, ^31^] In addition, The EPR signal of an alkoxy radical (Figure 2d) was detected in solution, which further verified the presence of radical species. The fact that the alkoxy radical is an isomer of the N‐centered radical can be informed from the spin density of the radical pair distributed on both oxygen atoms and nitrogen atoms (Figure 2e). The EPR signals of DMPOX and superoxide radical derive from the oxidation of DMPO and alkoxy radical. Besides, the alkoxy radical was also detected by the high‐resolution mass spectrum in positive ion mode with atmospheric pressure chemical ionization source (Figure 2f), [M+H]^+^ calcd. for [C_8_H_5_NO_2_]^+^: 147.0315, found: 147.0551. Therefore, the radical ion pair in the 4A2B crystal has been proved completely and the fluorescence at 400 nm and phosphorescence at 575 nm can be assigned to the radical ion pair.

**FIGURE 3:**
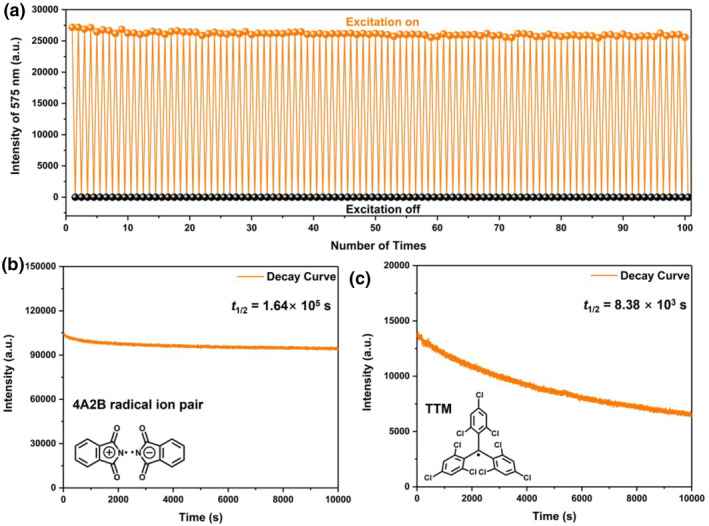
(a) The intensity of phosphorescence at 575 nm under 100 times on‐off excitation cycles. The photostability of (b) 4A2B crystal and (c) TTM@PMMA film, irradiated at 250 nm (5.5 mW) under air.

Homolysis of the N–N single bond is induced by photolysis, leading to the formation of an ion radical pair. The subsequent recombination of the ion radical pair is crucial for establishing a reversible light‐driven cycle for optical applications. As shown in Figure 3a, the phosphorescence of the ion radical pair (575 nm) denotes a remarkable consistency by a 100 times on‐off excitation cycles experiment. The relative mean deviation and relative standard deviation of these intensities at 575 nm are 1.28% and 0.94%, close to 0, revealing an outstanding repeatability of phosphorescence and a reversible transformation of the 4A2B molecule and radical ion pair. Hence, the steric hindrance provided by the crystal environment is used to restrict the distance of radical anion and cation for the reversible homolysis. As shown in Figure 3b, the radical ion pair of 4A2B demonstrates a great photostability under excitation at 250 nm with a half‐life of 1.64 × 10^5^ s, which is 20 times that of persistent radical TTM. Consequently, reversible homolysis serves to stabilize the radical ion pair against degradation, directly contributing to a longer functional lifetime in related devices.

**FIGURE 4:**
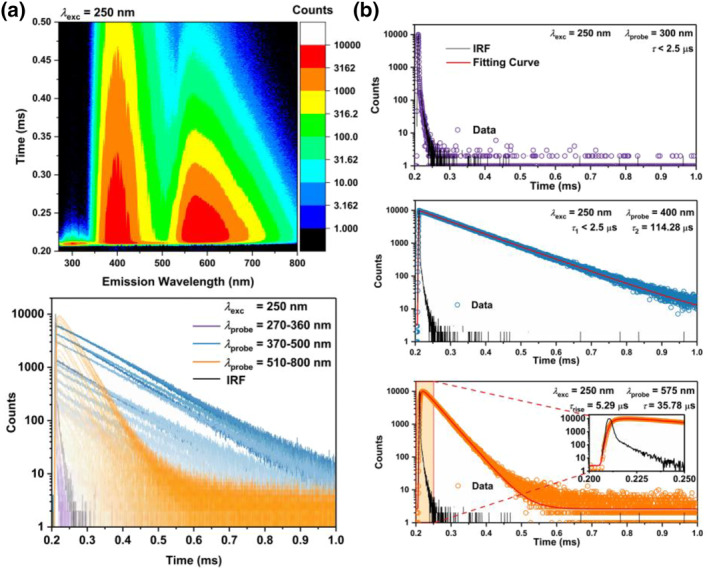
(a) Time‐resolved photoluminescence (TRPL) map and decay curves of the 4A2B crystal (*λ*_exc_ = 250 nm, *λ*_probe_ = 270–800 nm). (b) Kinetics at selected single wavelengths of the 4A2B crystal (*λ*_exc_ = 250 nm, *λ*_probe_ = 300 nm (top), 400 nm (middle) and 575 nm (bottom)).

The time‐resolved photoluminescence map and corresponding kinetics for the 4A2B crystal were collected to gain deep insight into the dynamics of dual emissions (Figure 4). Clearly, there are two prominent long‐lived decays at 400 and 575 nm as well as two short‐lived decays at 300 and 400 nm shown in Figure 4a. The lifetime probed at 300 nm (top of Figure 4b) is shorter than the instrument response function (IRF) of microsecond flash lamp (2.5 μs), which is assigned to the fluorescence of the 4A2B molecule. There are two components at 400 nm consisting of a long‐lived decay (*τ* = 114.28 μs, middle of Figure 4b) and a short‐lived decay less than IRF. Primarily, the short‐lived decay at 300 nm and the long‐lived decay at 400 nm are a pair of fluorescence and RTP emission assigned to the 4A2B molecule. Then, the kinetics at 575 nm exhibit a distinct rise (*τ*_rise_ = 5.29 μs, bottom of Figure 4b) followed by a long‐lived decay (*τ* = 35.78 μs) which is paired with the short‐lived decay at 400 nm as the RTP emission and fluorescence of the radical ion pair. This 5.29 μs rise is related to a structural change as the generation of radical ion pair.[^15^, ^42^, ^43^] Hence, there are two pairs of fluorescence and RTP emissions in the 4A2B crystal, which reveals its capacity for optical anti‐counterfeiting and validates this kind of molecular design for dual RTP emissions.

**FIGURE 5:**
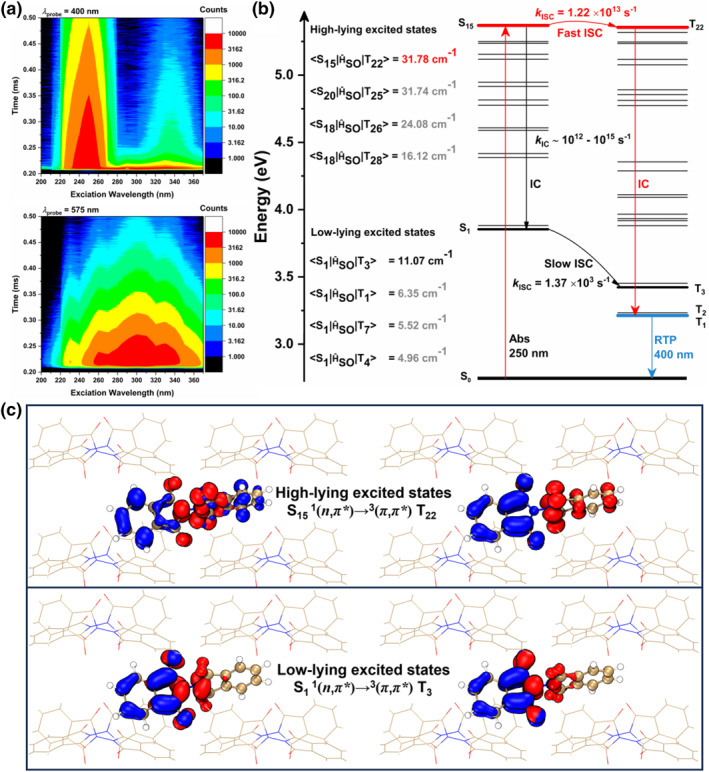
(a) Time‐resolved photoluminescence (TRPL) excitation map (*λ*_exc_ = 200–370 nm, *λ*_probe_ = 400 nm (top) and 575 nm (bottom)). (b) The spin‐orbital coupling matrix element (SOCME) of high‐lying and low‐lying excited states (left) and simplified excited states dynamics diagram of room‐temperature phosphorescence (RTP) emission at 400 nm (right). (c) The analysis for the distribution of the hole (red) and electron (blue) for S_1_, S_15_, T_3_ and T_22_ of the 4A2B molecule, isovalue: 0.005.

We further systematically investigated the excited state properties of these two RTP emissions. As shown in Figure 5a, the RTP at 400 nm has a strong response on the excitation at 250 nm, but the RTP emission at 575 nm is not. As is known to all, ISC is a vital process to RTP emission, which transfers singlet exciton into triplet exciton and *k*_ISC_ can be defined by the Fermi's golden rule[^44^, ^45^]

(1) $$k_{\text{ISC}}=\frac{2\pi }{\hbar }|<S_{n}\left|{Ĥ}_{\text{SO}}\right|T_{m}>{|}^{2}\rho_{\text{FC}}$$

where <*S*_*n*_|*Ĥ*_SO_|*T*_*m*_> denotes spin‐orbital coupling matrix element (SOCME), *ρ*_FC_ denotes the Franck‐Condon weighted density of states, *ħ* is the reduced Planck constant. Given that *k*_ISC_ is proportional to the square of the corresponding SOCME, which is often less than 1 cm^−1^ in organic systems, the SOCME between S_*n*_ and T_*m*_ (1 ≤ *n*, *m* ≤ 50) were calculated (Supporting Information S2; Excel S1). The singlet and triplet excited states with energy around 250 nm (4.96 ± 0.40 eV) were screened out (5 ≤ *n* ≤ 15, 13 ≤ *m* ≤ 22), whose SOCME over 15 cm^−1^ were attached on Figure 5b. The SOCME between S_1_ and T_m_ (1 ≤ *m* ≤ 10), over 1 cm^−1^, were also attached considering Kasha's rule. Franck‐Condon weighted density in Equation (1), another factor of ISC, is given by the semi‐classical Marcus theory as follow[^44^, ^45^]

(2) $$\rho_{\text{FC}}=\frac{1}{\sqrt{4\pi \lambda_{\text{re}}k_{B}T}}\exp\left(-\frac{{\left(\Delta E_{\text{ST}}\;-\;\lambda_{\text{re}}\right)}^{2}}{4\lambda_{\text{re}}k_{B}T}\right)$$

where *ρ*_FC_ is given as a function of the energy gap between singlet and triplet state (Δ*E*_ST_) as well as reorganization energy (*λ*_re_), *T* is temperature (300 K), *k*_B_ is Boltzmann constant. The singlet/triplet pairs of S_15_/T_22_ and S_1_/T_3_ were chosen to represent high‐lying and low‐lying excited states because of their highest SOCME. The introduction of high proportion n‐electron (n/π electron = 1) can increase the composition of ^1^(n, π*)→^3^(π, π*) transition (Figure 5c). Following El‐Sayed's rule, the *k*_ISC_ increases in the presence of two excited states with different natures. The *k*_ISC_ between S_15_ and T_22_ was calculated as 1.22 × 10^13^ s^−1^ but only 1.37 × 10^3^ s^−1^ between S_1_ and T_3_. The Δ*E*_ST_ and *λ*_re_ for calculation of *k*_ISC_ are listed in Supporting Information S1; Table S3. The high‐lying triplet excited state can be efficiently populated once *k*_ISC_ (S_15_→T_22_) is comparable to *k*_IC_ (10^12^∼10^15^ s^−1^), providing an efficient ISC channel to transfer excitons to the high‐lying triplet excited state.[^46^, ^47^, ^48^, ^49^] Therefore, the excitation at 250 nm can strongly generate the RTP emission at 400 nm.

**FIGURE 6:**
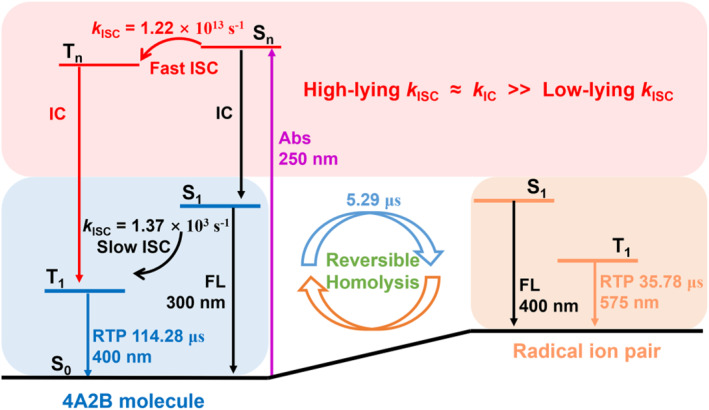
Simplified diagram of excited‐state dynamics in the 4A2B crystal.

The experimental results clearly demonstrated multiple emissions including two RTP emissions and two fluorescence of the 4A2B crystal. As summarized in Figure 6, under 250 nm excitation, the 4A2B molecule is excited to high‐lying singlet excited states. The *k*_ISC_ in high‐lying excited states (1.22 × 10^13^ s^−1^) is comparable to *k*_IC_ and much faster than *k*_ISC_ in low‐lying excited states (1.37 × 10^3^ s^−1^). Therefore, the excitons are mainly transferred to high‐lying triplet excited states leading to a RTP emission at 400 nm (*τ* = 114.28 μs). The 4A2B molecule at T_1_ state transfers to the radical ion pair within 5.29 μs, which radiates another RTP emission at 575 nm (*τ* = 35.78 μs). In addition to these two RTP emissions, there are two fluorescence radiated from the 4A2B molecule and radical ion pair, respectively.

Specifically, the dual RTP emissions at 400 and 575 nm, coupled with different response on excitation, allow for advanced multi‐level optical anti‐counterfeiting. For instance, specific patterns fabricated with the 4A2B crystal could remain invisible under ambient light or standard UV light, but reveal encrypted information only under 250 nm deep‐UV excitation due to the ultrafast ISC process. Furthermore, the excellent photostability (*t*_1/2_ = 1.64 × 10^5^ s) and reversibility over 100 cycles make this material suitable for long‐term operation. Additionally, the long‐lived lifetimes (114.28 μs) and the red region emission (575 nm) suggest great potential for high‐resolution time‐resolved bioimaging, allowing for the elimination of autofluorescence interference in biological tissues.

## 3 CONCLUSION

In this work, we had deeply investigated the dual RTP emissions of the 4A2B crystal. The high proportion of n‐electron of 4A2B enhances the SOC effect, thereby promoting both ISC and RTP efficiency. The RTP emission of the 4A2B molecule (400 nm, *τ* = 114.28 μs) has a strong response on excitation at 250 nm due to the exciton can be transferred to high‐lying triplet excited states effectively by an ultrafast ISC in high‐lying excited states (1.22 × 10^13^ s^−1^). Another RTP emission at 575 nm (*τ* = 35.78 μs) originates from the radical ion pair generated by the reversible homolysis of the N‐N single bond and this RTP emission exhibits great photostability with a half‐life of 1.64 × 10^5^ s and an outstanding repeatability with 100 times on‐off excitation cycles. There is a potential application in optical anti‐counterfeiting with these two RTP emissions as well as two normal fluorescence in the 4A2B crystal. Ultimately, our research elucidates the incorporation of organic molecules and radical ion pairs, thereby establishing a new method to achieve multiple emissions such as RTP emissions, fluorescence or TADF.

## CONFLICT OF INTEREST STATEMENT

The authors declare no conflicts of interest.

## ETHICS STATEMENT

No animal or human experiments were involved in this study.

## Supporting information

Supporting Information S1

Supporting Information S2

## Data Availability

The data that supports the findings of this study are available in the supplementary material of this article.
