# Supplementary material for: Dual room‐temperature phosphorescence derived from the reversible homolysis in the high proportion n‐electron organic crystal
Source: Smart Mol. 2026 Jul 21:e70083. Online ahead of print. doi: 10.1002/smo2.70083 (PMC13398727; doi:10.1002/smo2.70083)
Supplement: Supplementary file 1 — Supporting Information S1 [file SMO2-9999-0-s002.docx]

**Supporting Information**

Dual room-temperature phosphorescence derived from the reversible homolysis in the high proportion n-electron organic crystal

Junxiang Huang^1^, Dongqian Wang^1^, Dongping Wang^2^, Qichao Yao^3^, Xingyu Huangfu^1^, Guian Yang^1^, Saran Long*^1^, Jianjun Du^1^, Jiangli Fan^1^, Xiaojun Peng^1^

Junxiang Huang, Dongqian Wang, Xingyu Huangfu, Guian Yang, Saran Long, Jianjun Du, Jiangli Fan, Xiaojun Peng

^1^State Key Laboratory of Fine Chemicals, Frontiers Science Center for Smart Materials, Dalian University of Technology, Dalian 116024, China.
E-mail: srlong@dlut.edu.cn

Dongping Wang
^2^Chemistry Analysis & Research Center, School of Chemical Engineering, Dalian University of Technology, Dalian 116024, China.

Qichao Yao
^3^Shandong Laboratory of Advanced Materials and Green Manufacturing at Yantai, Yantai 264006, China

**Table of Contents**

Section 1. Materials, Synthesis and Measurements 3

Materials 3

Synthesis 3

Measurements 4

Single Crystal X-ray Diffraction (SC-XRD) Measurements 4

Powder X-ray Diffraction (PXRD) Measurements 4

Quantum Chemistry Calculations 4

Photophysical Measurements 5

Electron Paramagnetic Resonance (EPR) Measurements 5

Section 2. Scheme, Figures and Tables 7

Scheme 7

Scheme S1. The synthetic route of 4A2B. 7

Scheme S2. The synthetic route of TTM. 7

Figures 8

Figure S1. The schematic diagram of the high proportion of n-electrons. 8

Figure S2. HPLC spectrum recorded for 4A2B 9

Figure S3. ONIOM(QM1:QM2) model of 4A2B crystal. 10

Figure S4. The serial numbers of 4A2B molecule in crystal. 11

Figure S5. The hole-electron analysis for S_1_ and T_1_ of the multimer 12

Figure S6. The photoluminescence quantum yield of 4A2B crystal. 13

Figure S7. Molecular geometries of 4A2B molecule in S_1_/T_3_ and S_15_/T_22_ states. 14

Figure S8. EPR spectrum of TTM. 15

Figure S9. ^1^H NMR (500 MHz, 298 K, CD_2_Cl_2_) of 4A2B molecule. 16

Figure S10. ^13^C NMR (101 MHz, 298 K, DMSO-d6) of 4A2B molecule. 17

Tables 18

Table S1. TDDFT calculation results for 4A2B molecule and multimers.. 18

Table S2. Lattice parameters for 4A2B crystal. 19

Table S3. Parameters for calculation of intersystem crossing (ISC) rate. 20

REFERENCES 21

# **Section 1. Materials, Synthesis and Measurements**

## **Materials**

Phthalic anhydride, thiosemicarbazide, 1,3,5-Trichlorobenzene were purchased from Shanghai Bepharm Science&Technology Co.,Ltd.. Montmorillonite K-10 clay, anhydrous aluminum chloride, potassium tert-butoxide and tetrachloro-p-benzoquinone were purchased from Shanghai Aladdin Biochemical Technology Co.,Ltd.. Dichloromethane (Analytical Reagent, AR) and tetrahydrofuran (Analytical Reagent, AR) were purchased from Tianjin Damao Chemical Reagent Co., Ltd.. Tetrahydrofuran (High Performance Liquid Chromatography, HPLC) was purchased from Shanghai Annaiji Chemical Reagent, China. All reagents and solvents were used without further purification.

## **Synthesis**

***Synthesis of [2,2'-biisoindoline]-1,1',3,3'-tetraone (4A2B)***: A mixture of phthalic anhydride (2.2 mmol, 0.330 g), thiosemicarbazide (1 mmol, 0.091 g) and montmorillonite K-10 clay (1 g) was ground together properly and placed in a single-necked flask. The mixture was heated at 150°C under the nitrogen atmosphere for 12 hours. After the raw product was cooled down to the room temperature, the target product was extracted into dichloromethane (5×20 mL) and filtered off the solid. After the solvent was removed, the residue was purified using column chromatography on silica gel employing petroleum ether/dichloromethane (v:v = 3/1) and recrystallized in methanol and dichloromethane. The 4A2B crystal was further purified using a preparative liquid chromatograph (Shimadzu LC-20A Preparative Liquid Chromatograph using the column: 5 μm C18, 20×250 mm) with tetrahydrofuran (HPLC), as the eluent. The yield was 25%. ^1^H NMR (500 MHz, Methylene Chloride-*d*_2_) δ 7.99 (dd, *J* = 5.5, 3.1 Hz, 4H), 7.89 (dd, *J* = 5.5, 3.1 Hz, 4H). ^13^C NMR (101 MHz, DMSO-*d*_6_) δ 163.90, 136.77, 129.28, 125.29. HRMS-APCI (m/z): [M+H]^+^ calcd. for [C_16_H_9_N_2_O_4_]^+^, 293.0562; found, 293.0556.

***Synthesis of [tris(2,4,6-trichlorophenyl)methane] (HTTM)***: 1,3,5-Trichlorobenzene (6.1 g, 3.4 equiv., 33.5 mmol) and anhydrous aluminum chloride (2.0 g, 1.5equiv., 15.2 mmol) were added to a pressure vessel equipped with a magnetic stirrer. The vessel was purged with nitrogen before dry chloroform (0.8 mL, 1.0 equiv., 10 mmol) was added. The vessel was sealed, and the mixture was stirred at 95 ^o^C for 5 h. The vessel was cooled to room temperature before being depressurized. The contents were dumped into ice water, and the resulting mixture was extracted using dichloromethane and washed with brine. The combined organic fractions were dried over anhydrous sodium sulfate, and the removal of solvent from the resulting solution under reduced pressure gave the crude product HTTM as a white powder with a quantitative yield (4.0 g, 71%). HTTM was used in the subsequent steps without further purification.

***Synthesis of [tris(2,4,6-trichlorophenyl)methyl radical] (TTM)***: Under nitrogen atmosphere and in the dark, HTTM (2.20g, 3.97mmol) was dissolved in anhydrous tetrahydrofuran (50 mL), then, potassium tert-butoxide (0.67g, 5.94mmol) was added. After the mixture was stirred for 5 h at room temperature, tetrachloro-p-benzoquinone (2.63 g, 10.70 mmol) was added to the reaction mixture and allowed to react another 1 h. After reaction was finished, solvent was removed under vacuum. The resulting crude was purified by silica gel column chromatography using pure petroleum ether as the eluent, giving the pure TTM radical as a red powder (0.91 g, 41%). HRMS-APCI (m/z): [M]^-^ calcd. for [C_19_H_6_Cl_9_·]^-^, 552.7607; found, 552.7617.

## **Measurements**

### Single Crystal X-ray Diffraction (SC-XRD) Measurements

Single crystal of 4A2B suitable for the SC-XRD analysis was cultivated by slow solvent evaporation of purified 4A2B molecule in dichloromethane at room temperature for one week. Single crystal X-ray diffraction data of 4A2B was collected on a Bruker D8 Quest diffractometer using MoK radiation (*λ* = 0.71073 nm) source. The selected crystal was kept at 150.0 K during data collection. Using Olex2, ^1^ the structure was solved with the ShelXT3 structure solution program using Intrinsic Phasing and refined with the ShelXL4 refinement package using Least Squares minimization. ^2-5^ Selected crystal data are listed in Table S2. All crystallographic information in CIF format has been deposited at the Cambridge Crystallographic Data Center (CCDC) under deposition number 2513771 for 4A2B crystal via www.ccdc.cam.ac.uk/data_request/cif, or by emailing data_request@ccdc.cam.ac.uk, or by contacting the Cambridge Crystallographic Data Center, 12 Union Road, Cambridge CB2 1EZ, UK; fax: +44 1223 336033.

### Powder X-ray Diffraction (PXRD) Measurements

Crystal structure of samples was identified by the powder X-ray diffractometer (Rigaku SmartLab (9)) using CuKα radiation (*λ* = 0.15406 nm) at a tube-voltage of 45 kV and tube-current of 200 mA with a step size of 0.02^o^ and a scanning rate of 10^o^/min.

### Quantum Chemistry Calculations

The theoretical calculations were carried out with Gaussian 16 B.01^6^ and Orca 6.0.0 program package^7^. The quantum chemistry calculation of 4A2B crystal is considered by the ONIOM(QM1:QM2) ^8^ (our Own N-layer Integrated molecular Orbital molecular Mechanics) method. The computational model is constructed according to the SC-XRD data, and the detail packing structure is shown in Figure S3. In the ONIOM calculations, the cluster is divided into two layers: the innermost molecule (QM1 region, 30 atoms) is treated using accurate high-level quantum mechanics method (density functional theory, DFT), and the surrounding 8 molecules (QM2 region, 240 atoms) are processed using low-level quantum mechanics method. The QM2 region is treated using the PM7 method^9^ in Gaussian 16 B.01 program package and XTB2 method^10^ in ORCA 6.0.0 program package. The geometry optimization of excited states were performed by time-dependent density functional theory (TDDFT) calculations with PBE0^11^ density functional method and basis set def2-SVP^12^. The Grimme dispersion correction scheme D3BJ^13^ was added to consider weak dispersion interaction. The electronic properties were calculated with PBE0-D3BJ/def2-TZVP. The spin-orbital coupling (SOC) between S_n_ and T_m_ states were calculated by Orca 6.0.0. All SOCs were obtained at the TDDFT level of theory using the PBE0 functional and def2-TZVP basis set. The QM2 section was frozen during the geometry optimization for the S_n_ and T_m_ states. The calculation of potential energy surface (PES) of S_0_ and T_1_ were conducted by the broken-symmetry approach with UPBE0/def2-TZVP theory level. The spin density, hole-electron analysis, Mayer bond order were analyzed by Multiwfn 3.8(dev). ^14-17^ The images of molecules were generated using VMD 1.9.4a50. ^18^

### Photophysical Measurements

The UV-vis absorption spectrum was obtained on a Shimadzu UV-2600 spectrophotometer (Shimadzu, Japan) at room temperature. The photoluminescence spectra were measured on FLS 1000 (Edinburgh, UK) with continuous xenon lamp. The measurement temperature was controlled by a Liquid Nitrogen Cryostat from 100 K to 300 K. The vacuum environment of Liquid Nitrogen Cryostat was provided by a diaphragm pump, and pre-pumping was conducted for three hours prior to the test. The kinetics and time-resolved photoluminescence maps were also obtained by FLS 1000 with microsecond flash lamp. Photoluminescence quantum yield was obtained by HAMAMATSU PHOTONICS K.K C13534-31.

### Electron Paramagnetic Resonance (EPR) Measurements

EPR spectra were measured by Bruker E500 Spectrometer. For EPR spectra of 4A2B crystal, 10 mg 4A2B crystal was loaded into clean paramagnetic tube. The xenon lamp was used to provide full-spectrum light. For EPR spectra of 4A2B solution, 0.292 mg 4A2B crystal and 0.057 mg Dimethyl pyridine N-oxide (DMPO) were dissolved into 0.5 mL anhydrous tert-butylbenzene and deoxidized for 15 mins. The 4A2B solution with DMPO was irradiated with 365 nm light for 2 mins. For EPR spectra of TTM solution, 0.5 mg TTM was dissolved into 1 mL dichloromethane and measured without radical trap and light. EPR spectra were simulated using the Xepr software. ^19^

# **Section 2. Scheme, Figures and Tables**

## **Scheme**


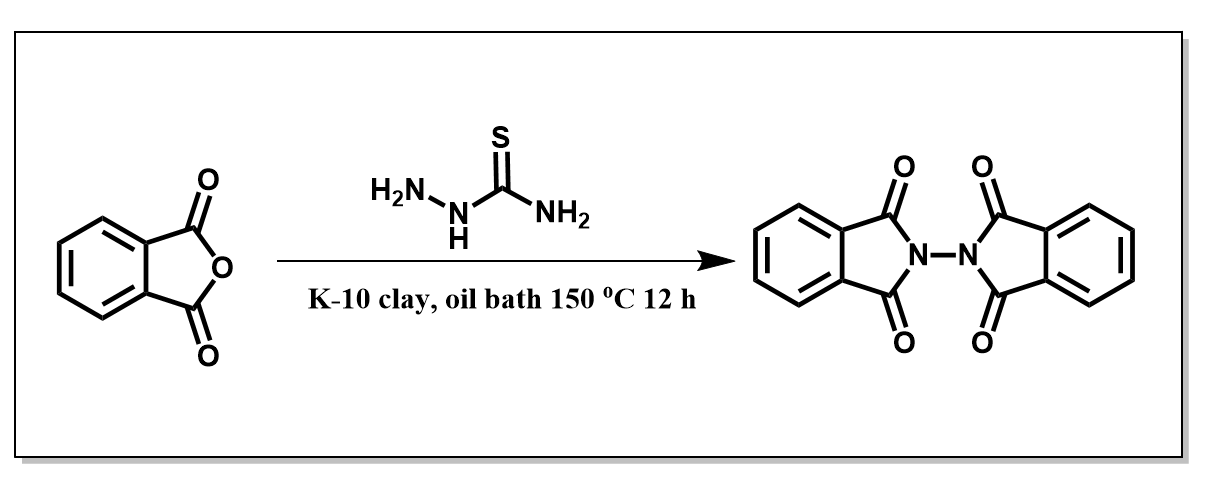
Scheme S1. The synthetic route of 4A2B.


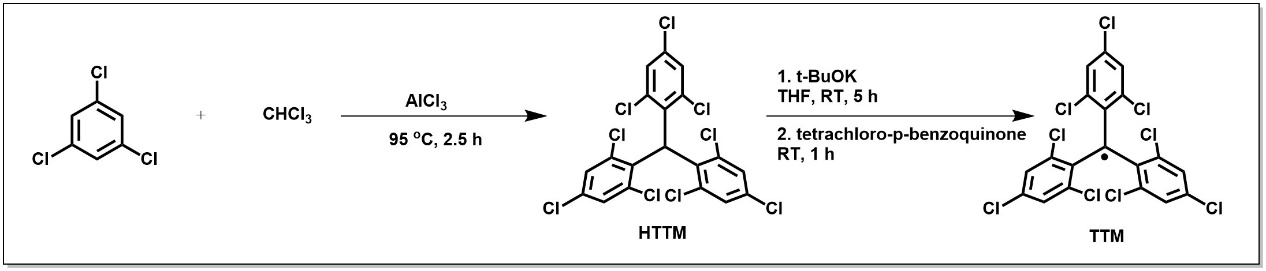
 Scheme S2. The synthetic route of TTM.

## **Figures**


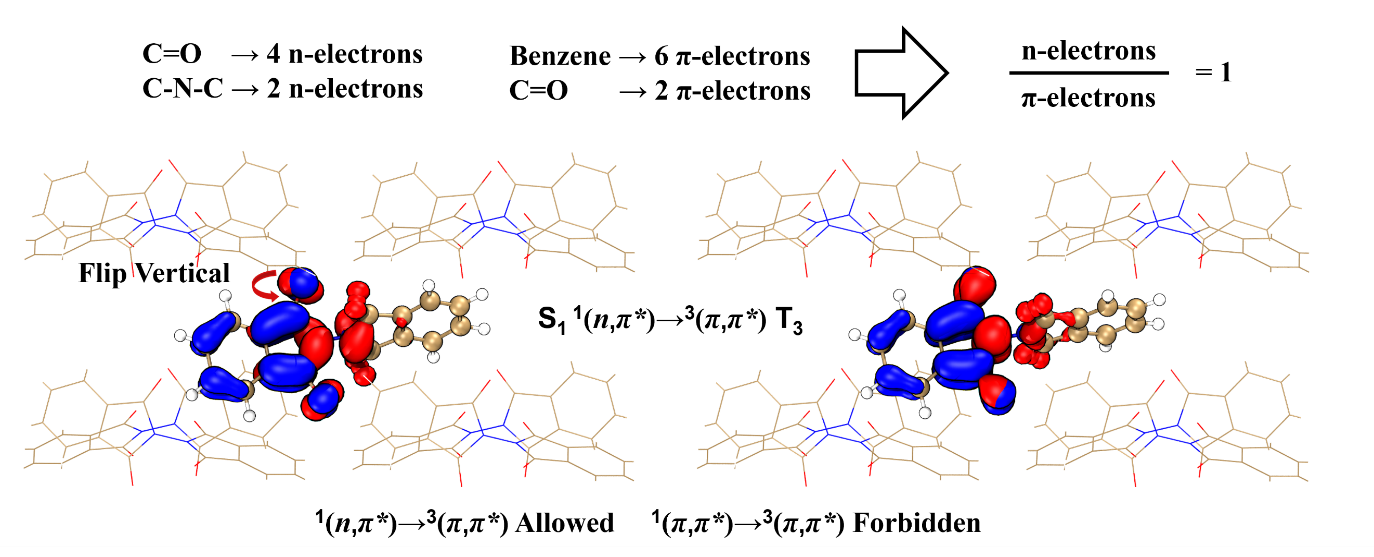


**Figure S1** The schematic diagram of the high proportion of n-electrons.





**Figure S2.** HPLC spectrum recorded for 4A2B, measured by Shimadzu LC-2050C 3D Liquid Chromatograph using the column: ShimNex CS C18, 5 μm, 4.6×150 mm with tetrahydrofuran as the eluent under 1.0 mL·min-1 speed, 25 ^o^C.


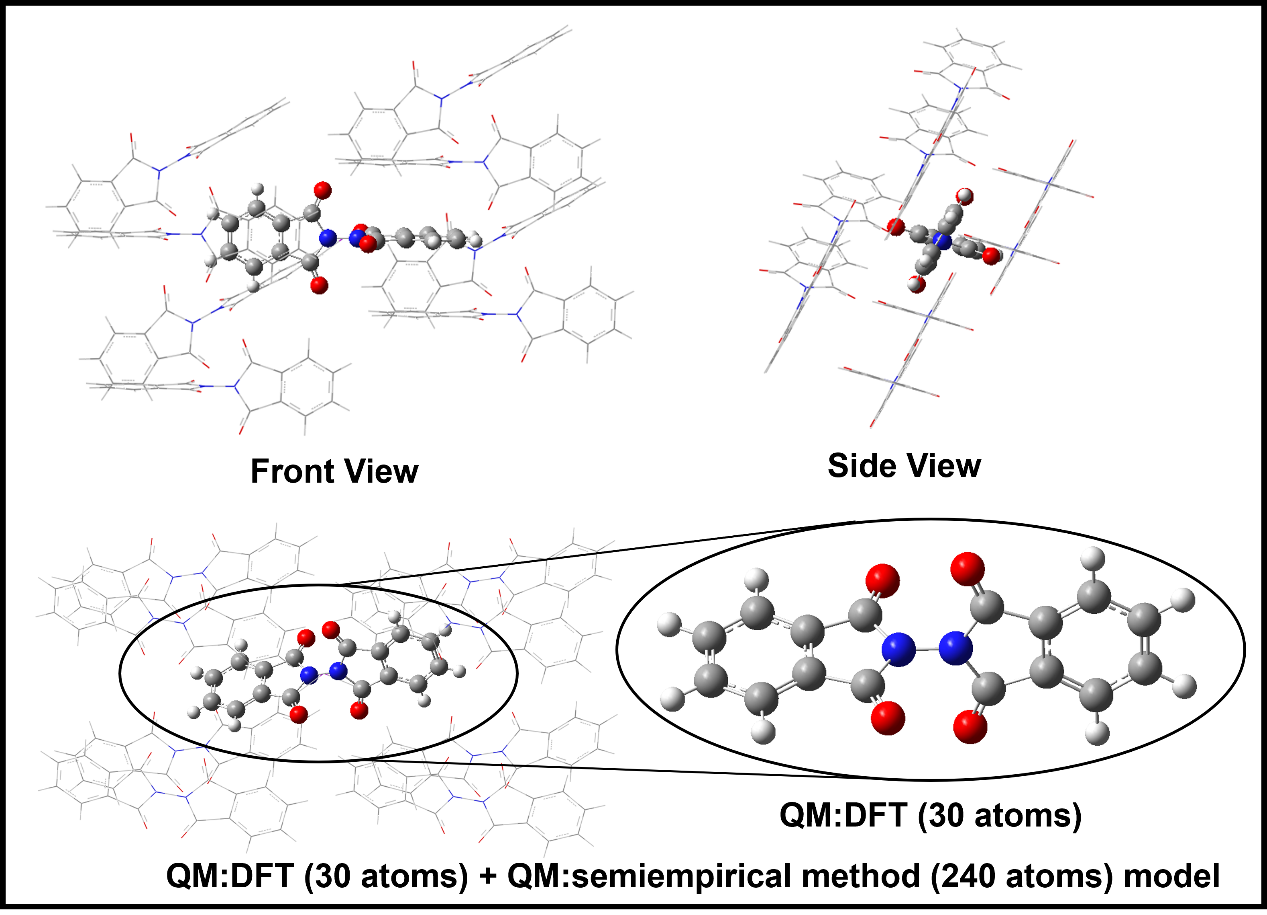


**Figure S3**. ONIOM(QM1:QM2) model of 4A2B crystal.


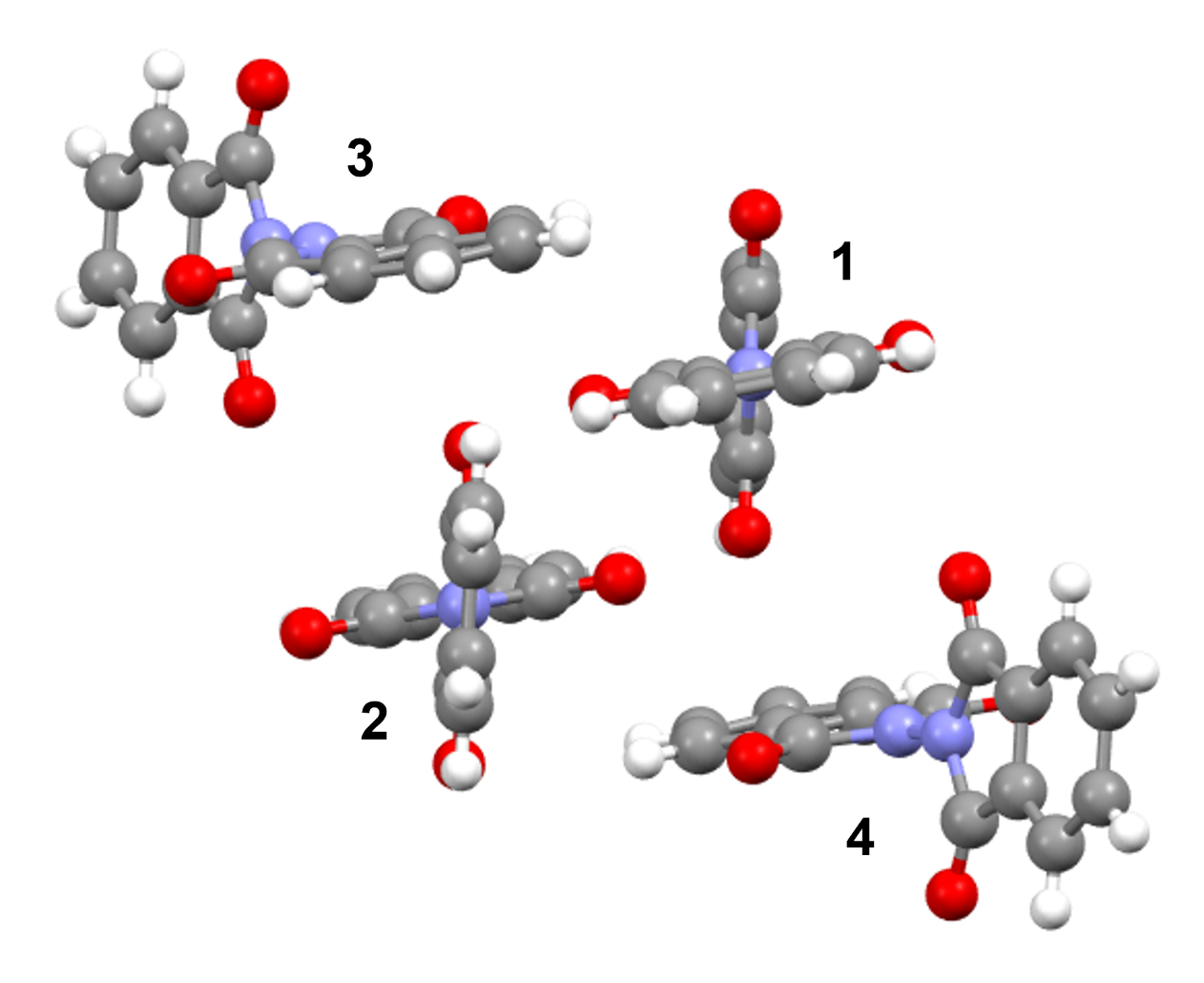


**Figure S4.** The serial numbers of 4A2B molecule in crystal. For example, No.1 and No.2 4A2B molecules can be considered as 12 dimer, No.1, No.2 and No.3 4A2B molecules can be considered as 123 trimer, all four 4A2B molecules can be considered as 1234 tetramer.


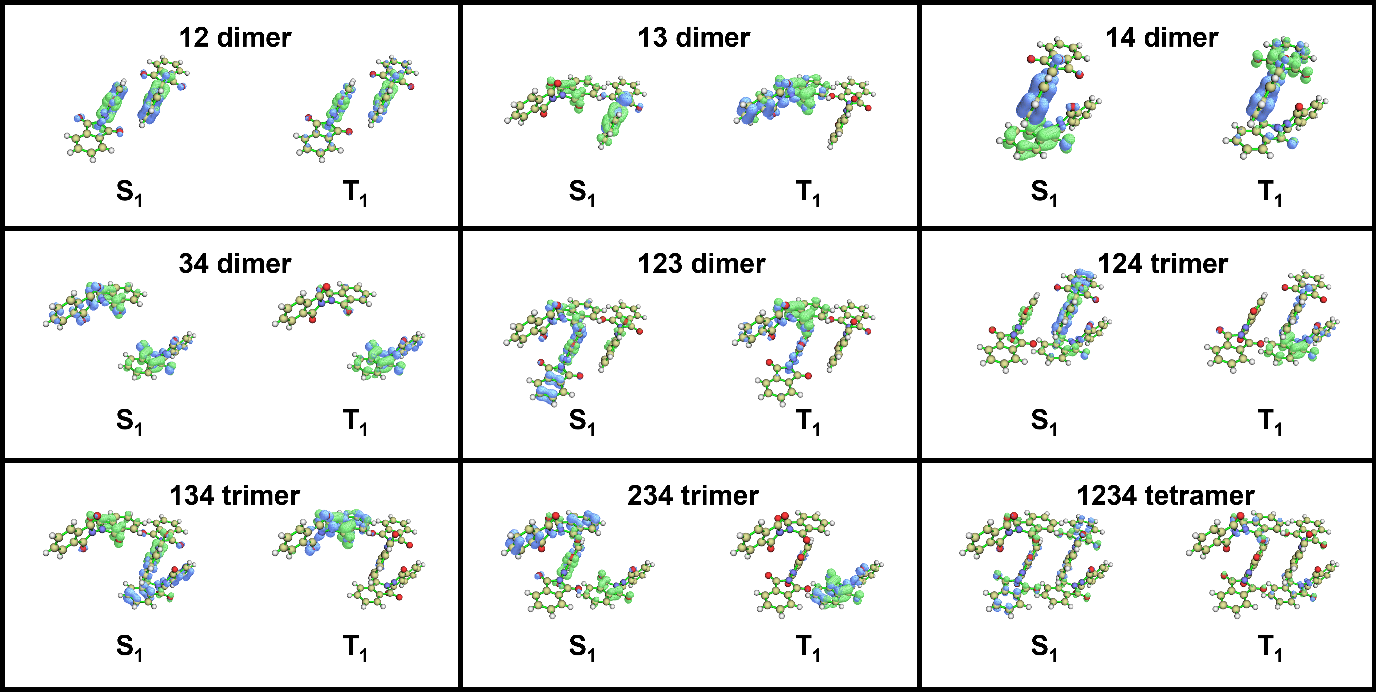


**Figure S5** The analysis for the distribution of the hole (blue) and electron (hole) for S_1_ and T_1_ of the multimer, isovalue: 0.002.





**Figure S6.** The photoluminescence quantum yield of 4A2B crystal.


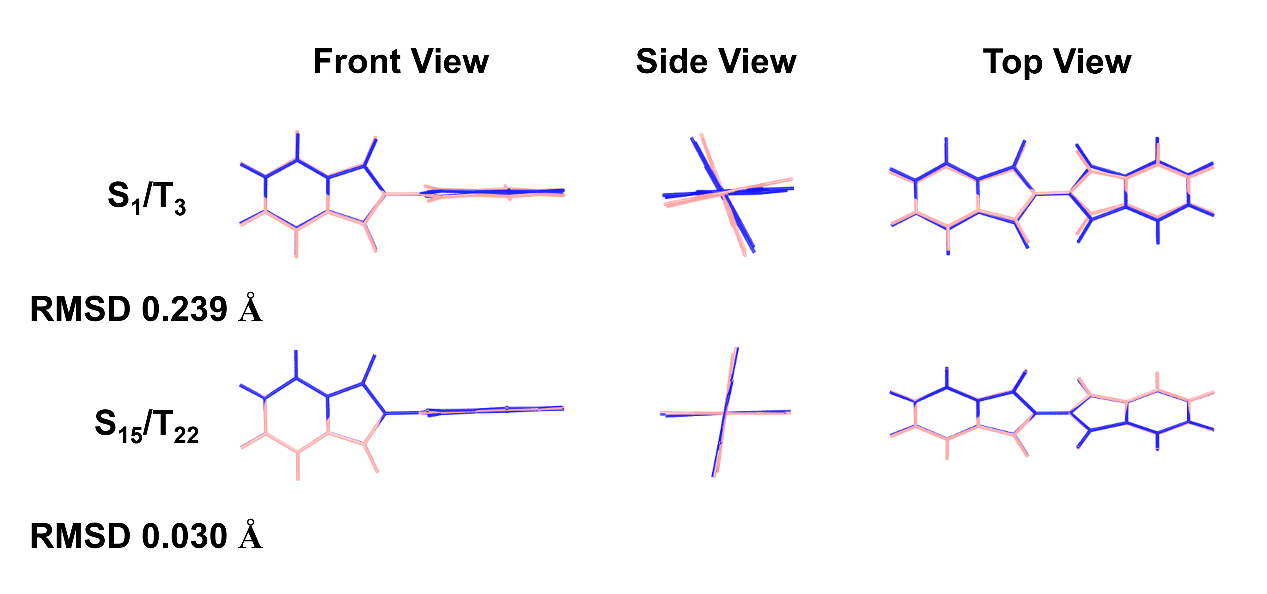


**Figure S7.** Molecular geometries of 4A2B molecule in S_1_/T_3_ and S_15_/T_22_ states with the RMSD values referenced to the singlet state, blue is singlet state, pink is triplet state.





**Figure S8.** EPR spectrum of TTM in dichloromethane (1×10^3^ mol/L) without light, g = 2.0000.


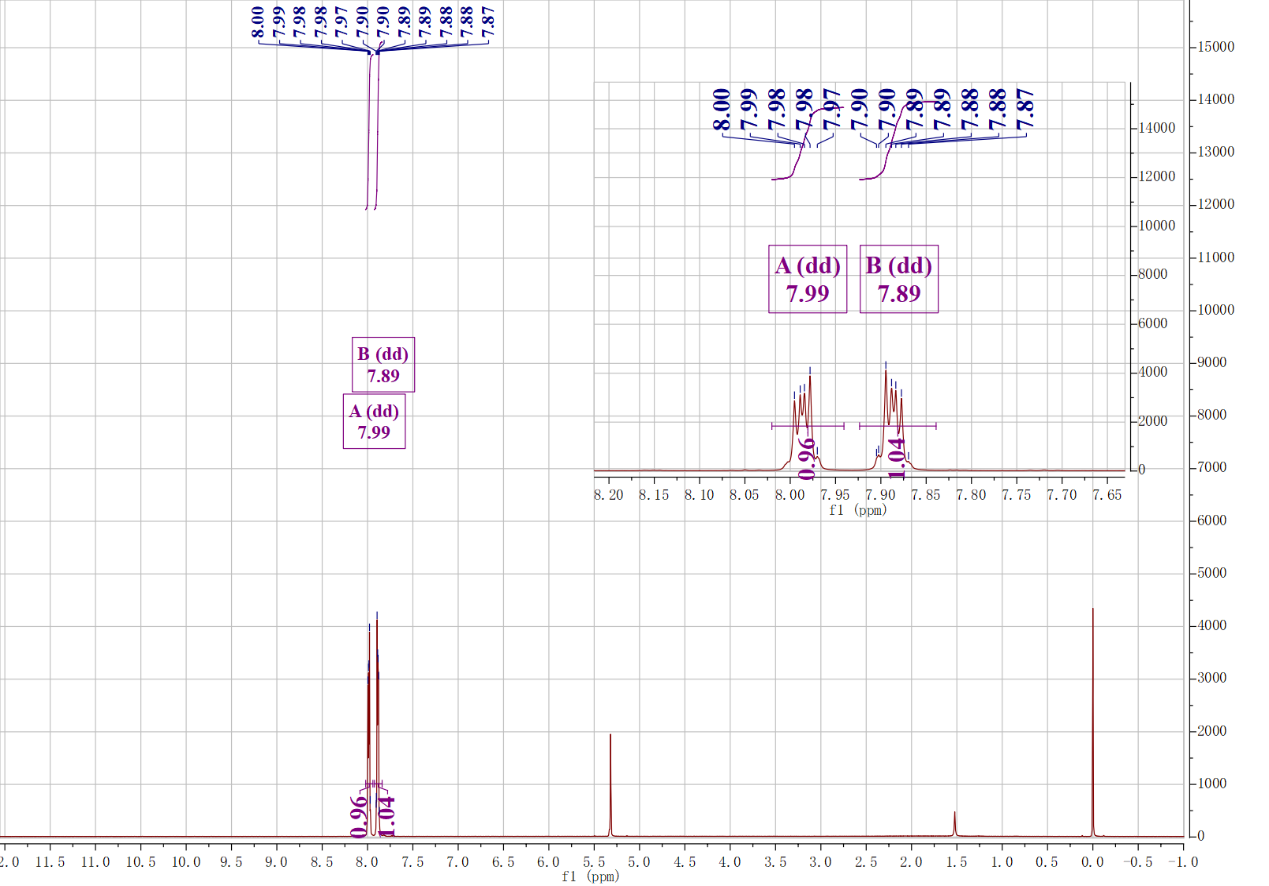


**Figure S9.** ^1^H NMR (500 MHz, 298 K, CD_2_Cl_2_) of 4A2B molecule.


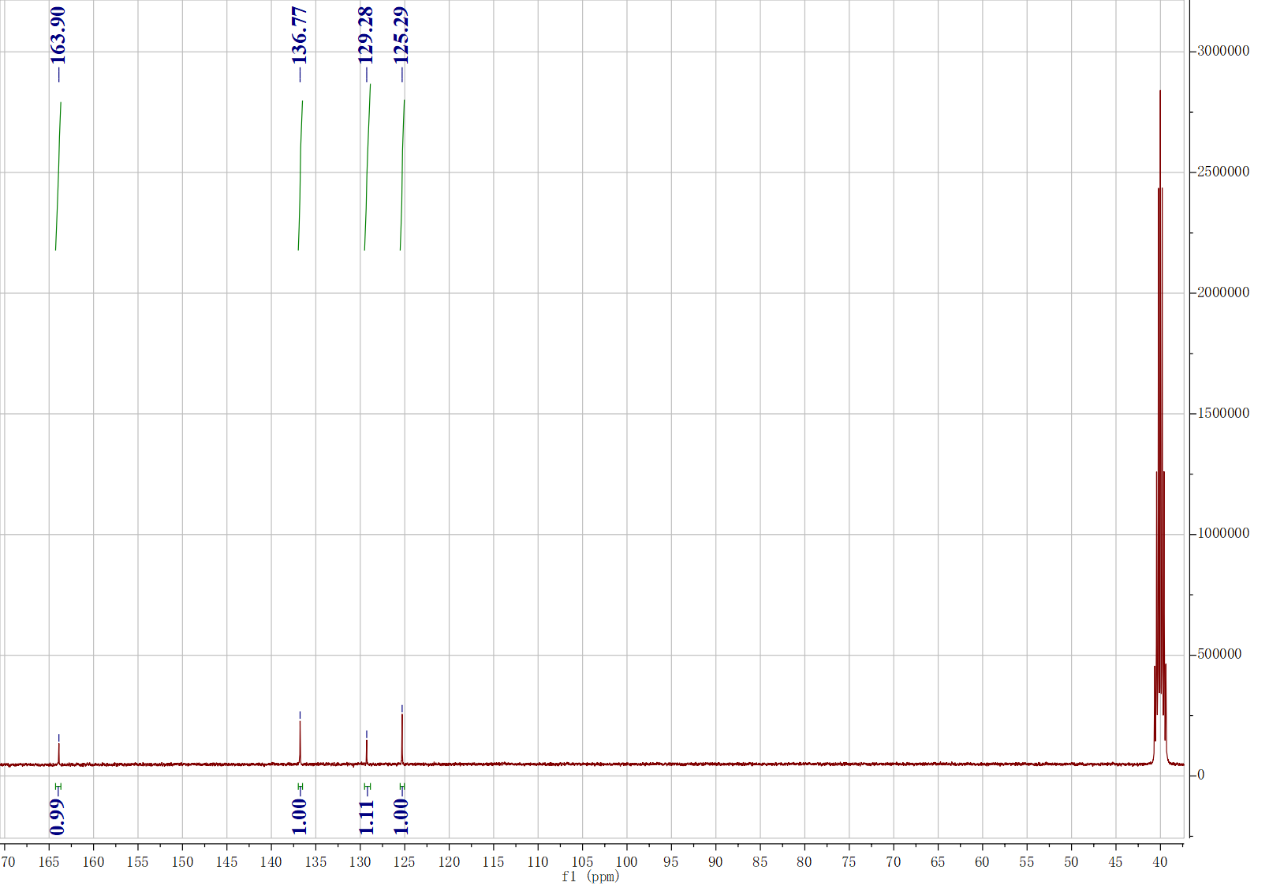


**Figure S10.** ^13^C NMR (101 MHz, 298 K, DMSO-d6) of 4A2B molecule.

## **Tables**

| Table S1. TDDFT calculation results for 4A2B molecule and multimers (The serial numbers of 4A2B molecule in crystal are shown in Figure S3). | | |
| --- | --- | --- |
|  | S_1_→S_0_/eV | T_1_→S_0_/eV |
| Monomer | 4.13 | 3.17 |
| 12 Dimer | 4.00 | 3.09 |
| 13 Dimer | 3.99 | 3.14 |
| 14 Dimer | 3.98 | 3.13 |
| 34 Dimer | 3.98 | 3.14 |
| 123 Trimer | 4.00 | 3.14 |
| 124 Trimer | 3.98 | 3.14 |
| 234 Trimer | 3.98 | 3.13 |
| 134 Trimer | 3.98 | 3.13 |
| 1234 Tetramer | 3.98 | 3.14 |

| Table S2. Lattice parameters for 4A2B crystal. | |
| --- | --- |
| Sample | 4A2B |
| Crystal system | monoclinic |
| Space group | *P*2_1_/c |
| Lattice parameters | *a =* 8.489(2) |
|  | *b* = 13.401(4) |
|  | *c* = 11.485(3) |
|  | α = 90 |
|  | β = 92.226(9) |
|  | γ = 90 |
| Unit cell volume | 1305.5(6) |
| Measurement temperature | 150(2) K |
| Goodness of fit | 1.054 |
| R_1_ | 4.48% |
| wR_2_ | 12.07% |

| Table S3. Parameters for calculation of intersystem crossing (ISC) rate. | | | | |
| --- | --- | --- | --- | --- |
| S_n_/T_m_ | *λ*/eV | Δ*E*_SnTm_/eV | <T_m_\|H_SO_\|S_n_>/cm^-1^ | *k*_ISC_/s^-1^ |
| S_1_/T_3_ | 0.33 | 0.06 | 11.07 | 1.37 × 10^3^ |
| S_15_/T_22_ | 0.05 | 0.02 | 31.78 | 1.22 × 10^13^ |

## **REFERENCES**

[1] Dolomanov, O. V.; Bourhis, L. J.; Gildea, R. J.; Howard, J. A. K.; Puschmann, H. *OLEX2*: a complete structure solution, refinement and analysis program. *J.* *Appl. Cryst.* **2009**, *42*, 339-341.

[2] Sheldrick, G. M. Program for crystal-structure refinement. University of Göttingen*,* **1997**.

[3] Sheldrick, G. M. A short history of SHELX. *Acta Crystallogr., Sect. A:Found. Crystallogr.* **2008**, *64*, 112-122.

[4] Sheldrick, G. M. Crystal structure refinement with SHELXL. *Acta Crystallogr., Sect. C:Cryst. Struct. Commun.* **2015**, *71*, 3-8.

[5] Lübben, J.; Wandtke, C. M.; Hübschle, C. B.; Ruf, M.; Sheldrick, G. M.; Dittrich, B. Aspherical scattering factors for SHELXL–model, implementation and application. *Acta Crystallogr., Sect. A:Found. Crystallogr.* **2019**, *75*, 50-62.

[6] Frisch, M. J.; Trucks, G. W.; Schlegel, H. B.; Scuseria, G. E.; Robb, M. A.; Cheeseman, J. R.; Scalmani, G.; Barone, V. P. G. A.; Petersson, G. A.; Nakatsuji, H. J. W. C.; Li, X. Gaussian 16. Gaussian, Inc. Wallingford, CT: **2016**.

[7] Neese, F. The ORCA program system. *Wiley Interdiscip. Rev.:Comput. Mol. Sci.* **2012**, *2*, 73-78.

[8] Humbel, S.; Sieber, S.; Morokuma, K. The IMOMO method: Integration of different levels of molecular orbital approximations for geometry optimization of large systems: Test for *n*‐butane conformation and *S_N_*2 reaction: RCl^+^ Cl^−^. *J. Chem. Phys.* **1996**, *105*, 1959-1967.

[9] Stewart, J. J. Optimization of parameters for semiempirical methods VI: more modifications to the NDDO approximations and re-optimization of parameters. *J. Mol. Model.* **2013**, *19*, 1-32.

[10] Koopman, J.; Grimme, S. Calculation of electron ionization mass spectra with semiempirical GFNn-xTB methods. *ACS omega* **2019**, *4*, 15120-15133.

[11] Adamo, C.; Barone, V. Toward reliable density functional methods without adjustable parameters: The PBE0 model. *J. Chem. phys.* **1999**, *110*, 6158-6170.

[12] Chen, Y.; Chen, B.; Sun, L.; Leng, W.; Meng, Y.; Gu, C.; Chen, Y.; Song, T.; Huang, J.; Wang, J.; Wang, G.; Shen, X.; Rao, F. Nano-composite phase-change antimony thin film for fast and persistent memory operations Mater. Today Phys. **2022**, *22*, 100584.

[13] Zhao, Y.; Truhlar, D. G. Density functional for spectroscopy: no long-range self-interaction error, good performance for Rydberg and charge-transfer states, and better performance on average than B3LYP for ground states. *J. Phys. Chem. A* **2006**, *110*, 13126-13130.

[14] Lu, T. A comprehensive electron wavefunction analysis toolbox for chemists, Multiwfn. *J. Chem. Phys.* **2024**, *161*, 082503.

[15] Lu, T.; Chen, F. W. Multiwfn: A multifunctional wavefunction analyzer. *J. Comput. Chem.* **2012**, *33*, 580-592.

[16] Liu, Z.; Lu, T.; Chen, Q. An sp-hybridized all-carboatomic ring, cyclo[18] carbon: Electronic structure, electronic spectrum, and optical nonlinearity. *Carbon* **2020**, *165*, 461-467.

[17] Mayer, I. Charge, bond order and valence in the AB initio SCF theory. *Chem. Phys. Lett.* **1983**, *97*, 270-274.

[18] Humphrey, W.; Dalke, A.; Schulten, K. VMD: visual molecular dynamics. *J. Mole. Graphics* **1996**, *14*, 33-38.

[19] Hanson, G. R.; Gates, K. E.; Noble, C. J.; Mitchell, A.; Benson, S.; Griffin, M.; Burrage, K. XSophe—Sophe—XeprView: A computer simulation software suite for the analysis of continuous wave EPR spectra. *In* *EPR of free radicals in solids: Trends in methods and applications*, Springer, **2003**; pp 197-237.
